# Supplementary material for: Beneficial Effects of Astragalus membranaceus (Fisch.) Bunge Extract in Controlling Inflammatory Response and Preventing Asthma Features
Source: Int J Mol Sci. 2023 Jun 30;24(13):10954. doi: 10.3390/ijms241310954 (PMC10342042; doi:10.3390/ijms241310954)
Supplement: Supplementary file 1 [file ijms-24-10954-s001.zip › ijms-2443608-supplementary.pptx]

## Slide 1
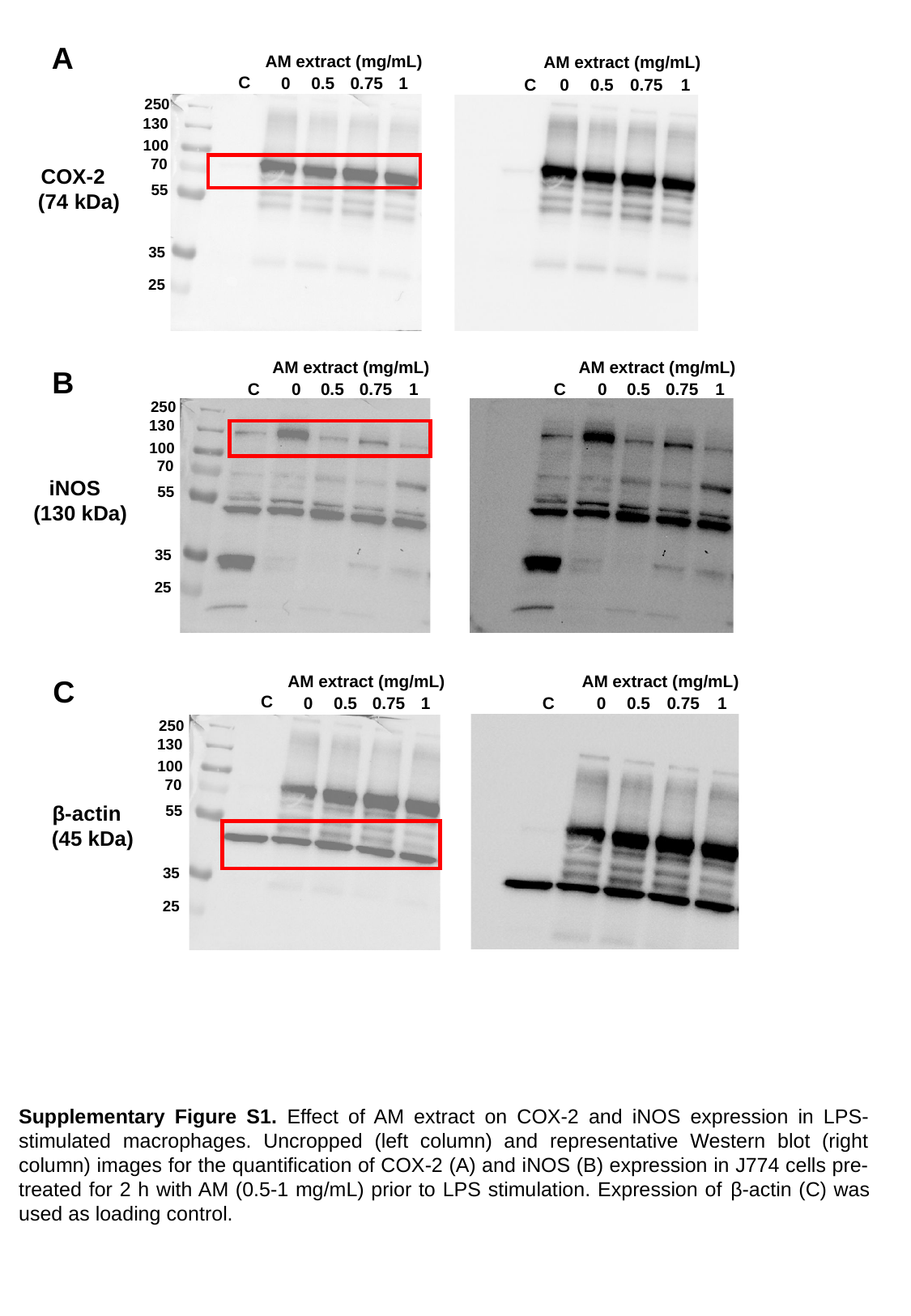

A
AM extract (mg/mL)
C
0.75
0.5
0
1
AM extract (mg/mL)
C
0
0.5
0.75
1
250
130
100
70
55
35
25
COX-2
(74 kDa)
AM extract (mg/mL)
0.75
0.5
0
1
C
AM extract (mg/mL)
0.75
0.5
0
1
C
250
130
100
70
55
35
25
iNOS
(130 kDa)
B
AM extract (mg/mL)
C
0.75
0.5
0
1
AM extract (mg/mL)
C
0
0.5
0.75
1
250
130
100
70
55
35
25
β-actin
(45 kDa)
C
Supplementary Figure S1. Effect of AM extract on COX-2 and iNOS expression in LPS-stimulated macrophages. Uncropped (left column) and representative Western blot (right column) images for the quantification of COX-2 (A) and iNOS (B) expression in J774 cells pre-treated for 2 h with AM (0.5-1 mg/mL) prior to LPS stimulation. Expression of β-actin (C) was used as loading control.

## Slide 2
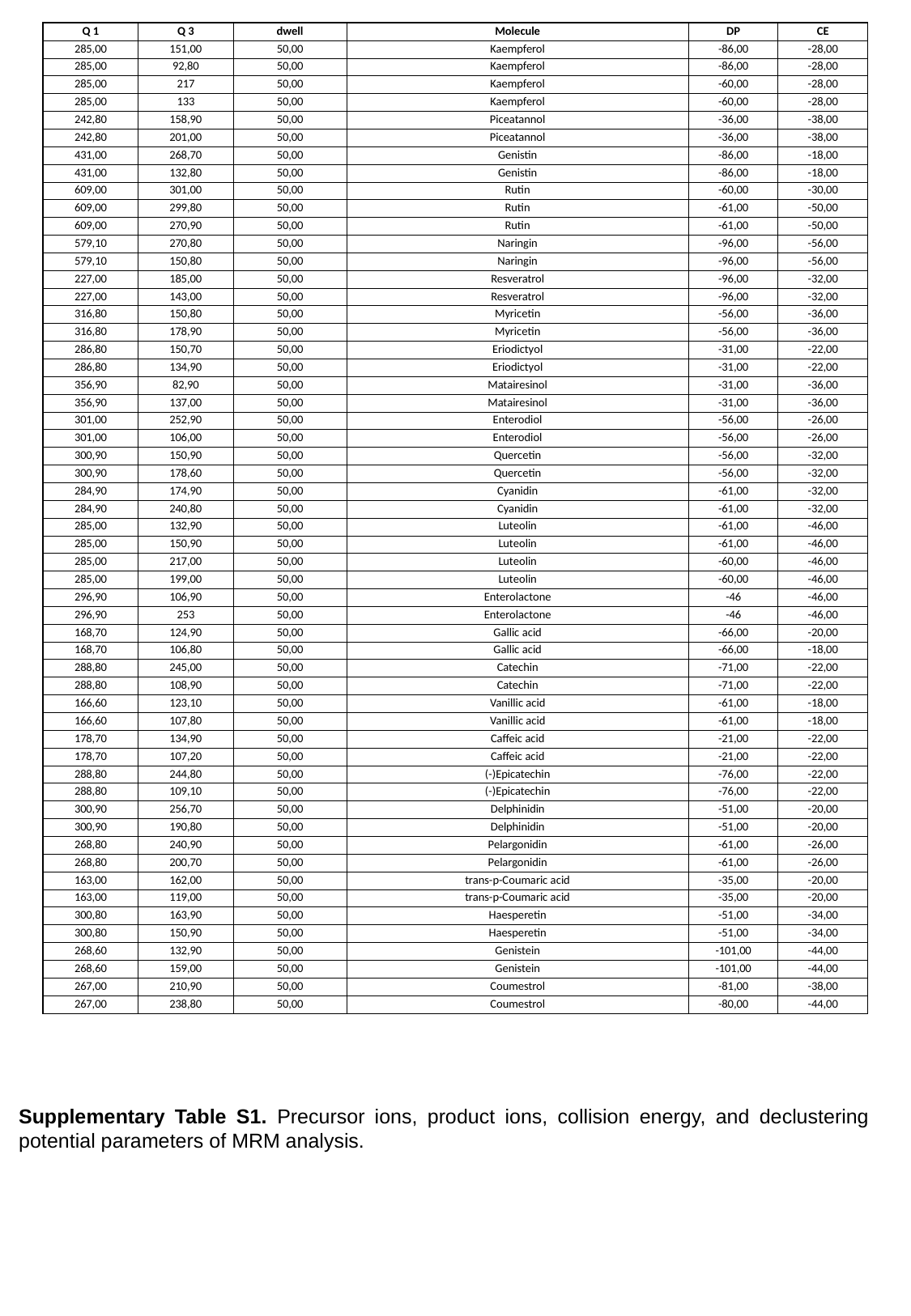

| Q 1 | Q 3 | dwell | Molecule | DP | CE |
| --- | --- | --- | --- | --- | --- |
| 285,00 | 151,00 | 50,00 | Kaempferol | -86,00 | -28,00 |
| 285,00 | 92,80 | 50,00 | Kaempferol | -86,00 | -28,00 |
| 285,00 | 217 | 50,00 | Kaempferol | -60,00 | -28,00 |
| 285,00 | 133 | 50,00 | Kaempferol | -60,00 | -28,00 |
| 242,80 | 158,90 | 50,00 | Piceatannol | -36,00 | -38,00 |
| 242,80 | 201,00 | 50,00 | Piceatannol | -36,00 | -38,00 |
| 431,00 | 268,70 | 50,00 | Genistin | -86,00 | -18,00 |
| 431,00 | 132,80 | 50,00 | Genistin | -86,00 | -18,00 |
| 609,00 | 301,00 | 50,00 | Rutin | -60,00 | -30,00 |
| 609,00 | 299,80 | 50,00 | Rutin | -61,00 | -50,00 |
| 609,00 | 270,90 | 50,00 | Rutin | -61,00 | -50,00 |
| 579,10 | 270,80 | 50,00 | Naringin | -96,00 | -56,00 |
| 579,10 | 150,80 | 50,00 | Naringin | -96,00 | -56,00 |
| 227,00 | 185,00 | 50,00 | Resveratrol | -96,00 | -32,00 |
| 227,00 | 143,00 | 50,00 | Resveratrol | -96,00 | -32,00 |
| 316,80 | 150,80 | 50,00 | Myricetin | -56,00 | -36,00 |
| 316,80 | 178,90 | 50,00 | Myricetin | -56,00 | -36,00 |
| 286,80 | 150,70 | 50,00 | Eriodictyol | -31,00 | -22,00 |
| 286,80 | 134,90 | 50,00 | Eriodictyol | -31,00 | -22,00 |
| 356,90 | 82,90 | 50,00 | Matairesinol | -31,00 | -36,00 |
| 356,90 | 137,00 | 50,00 | Matairesinol | -31,00 | -36,00 |
| 301,00 | 252,90 | 50,00 | Enterodiol | -56,00 | -26,00 |
| 301,00 | 106,00 | 50,00 | Enterodiol | -56,00 | -26,00 |
| 300,90 | 150,90 | 50,00 | Quercetin | -56,00 | -32,00 |
| 300,90 | 178,60 | 50,00 | Quercetin | -56,00 | -32,00 |
| 284,90 | 174,90 | 50,00 | Cyanidin | -61,00 | -32,00 |
| 284,90 | 240,80 | 50,00 | Cyanidin | -61,00 | -32,00 |
| 285,00 | 132,90 | 50,00 | Luteolin | -61,00 | -46,00 |
| 285,00 | 150,90 | 50,00 | Luteolin | -61,00 | -46,00 |
| 285,00 | 217,00 | 50,00 | Luteolin | -60,00 | -46,00 |
| 285,00 | 199,00 | 50,00 | Luteolin | -60,00 | -46,00 |
| 296,90 | 106,90 | 50,00 | Enterolactone | -46 | -46,00 |
| 296,90 | 253 | 50,00 | Enterolactone | -46 | -46,00 |
| 168,70 | 124,90 | 50,00 | Gallic acid | -66,00 | -20,00 |
| 168,70 | 106,80 | 50,00 | Gallic acid | -66,00 | -18,00 |
| 288,80 | 245,00 | 50,00 | Catechin | -71,00 | -22,00 |
| 288,80 | 108,90 | 50,00 | Catechin | -71,00 | -22,00 |
| 166,60 | 123,10 | 50,00 | Vanillic acid | -61,00 | -18,00 |
| 166,60 | 107,80 | 50,00 | Vanillic acid | -61,00 | -18,00 |
| 178,70 | 134,90 | 50,00 | Caffeic acid | -21,00 | -22,00 |
| 178,70 | 107,20 | 50,00 | Caffeic acid | -21,00 | -22,00 |
| 288,80 | 244,80 | 50,00 | (-)Epicatechin | -76,00 | -22,00 |
| 288,80 | 109,10 | 50,00 | (-)Epicatechin | -76,00 | -22,00 |
| 300,90 | 256,70 | 50,00 | Delphinidin | -51,00 | -20,00 |
| 300,90 | 190,80 | 50,00 | Delphinidin | -51,00 | -20,00 |
| 268,80 | 240,90 | 50,00 | Pelargonidin | -61,00 | -26,00 |
| 268,80 | 200,70 | 50,00 | Pelargonidin | -61,00 | -26,00 |
| 163,00 | 162,00 | 50,00 | trans-p-Coumaric acid | -35,00 | -20,00 |
| 163,00 | 119,00 | 50,00 | trans-p-Coumaric acid | -35,00 | -20,00 |
| 300,80 | 163,90 | 50,00 | Haesperetin | -51,00 | -34,00 |
| 300,80 | 150,90 | 50,00 | Haesperetin | -51,00 | -34,00 |
| 268,60 | 132,90 | 50,00 | Genistein | -101,00 | -44,00 |
| 268,60 | 159,00 | 50,00 | Genistein | -101,00 | -44,00 |
| 267,00 | 210,90 | 50,00 | Coumestrol | -81,00 | -38,00 |
| 267,00 | 238,80 | 50,00 | Coumestrol | -80,00 | -44,00 |
Supplementary Table S1. Precursor ions, product ions, collision energy, and declustering potential parameters of MRM analysis.

## Slide 3
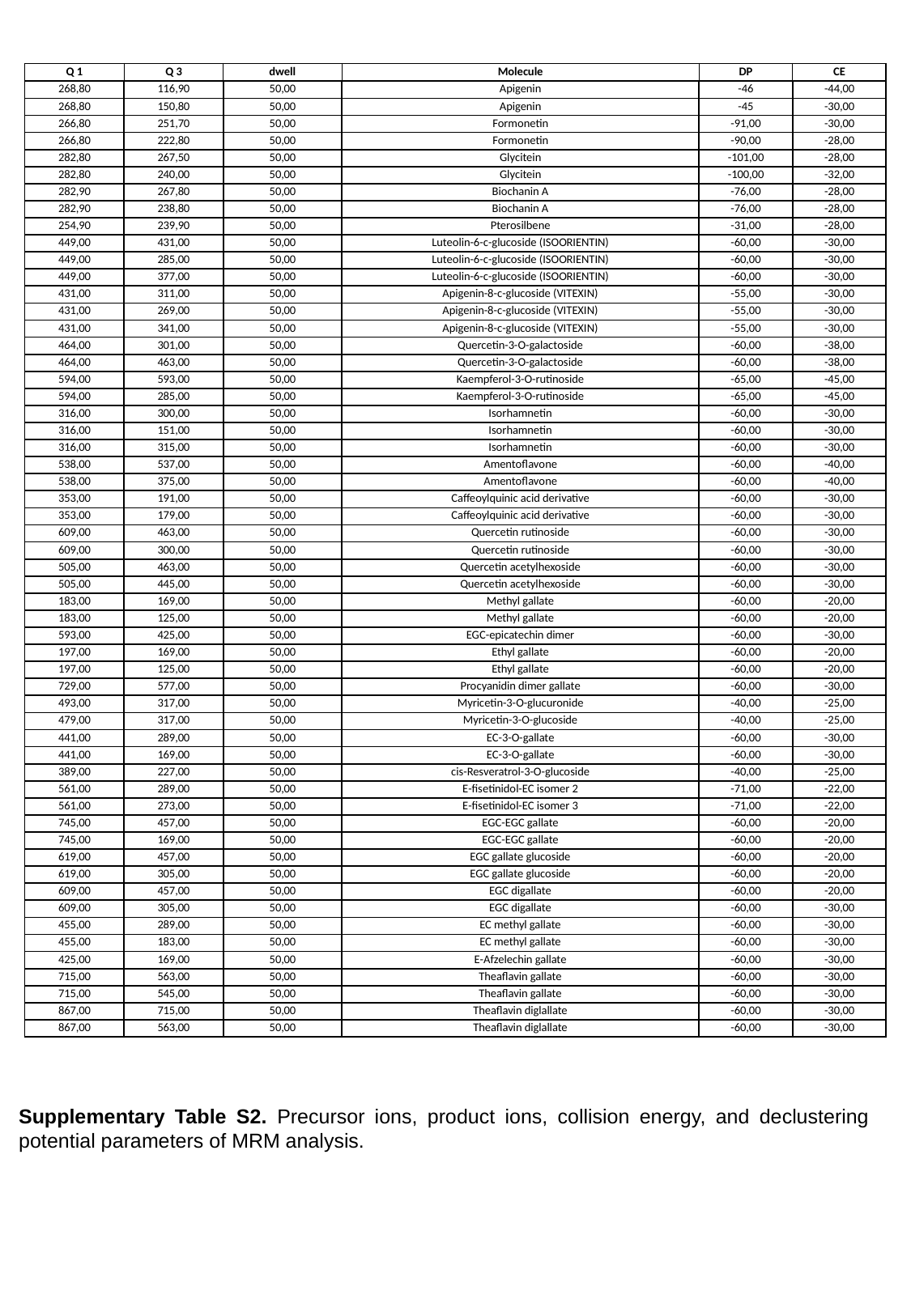

| Q 1 | Q 3 | dwell | Molecule | DP | CE |
| --- | --- | --- | --- | --- | --- |
| 268,80 | 116,90 | 50,00 | Apigenin | -46 | -44,00 |
| --- | --- | --- | --- | --- | --- |
| 268,80 | 150,80 | 50,00 | Apigenin | -45 | -30,00 |
| 266,80 | 251,70 | 50,00 | Formonetin | -91,00 | -30,00 |
| 266,80 | 222,80 | 50,00 | Formonetin | -90,00 | -28,00 |
| 282,80 | 267,50 | 50,00 | Glycitein | -101,00 | -28,00 |
| 282,80 | 240,00 | 50,00 | Glycitein | -100,00 | -32,00 |
| 282,90 | 267,80 | 50,00 | Biochanin A | -76,00 | -28,00 |
| 282,90 | 238,80 | 50,00 | Biochanin A | -76,00 | -28,00 |
| 254,90 | 239,90 | 50,00 | Pterosilbene | -31,00 | -28,00 |
| 449,00 | 431,00 | 50,00 | Luteolin-6-c-glucoside (ISOORIENTIN) | -60,00 | -30,00 |
| 449,00 | 285,00 | 50,00 | Luteolin-6-c-glucoside (ISOORIENTIN) | -60,00 | -30,00 |
| 449,00 | 377,00 | 50,00 | Luteolin-6-c-glucoside (ISOORIENTIN) | -60,00 | -30,00 |
| 431,00 | 311,00 | 50,00 | Apigenin-8-c-glucoside (VITEXIN) | -55,00 | -30,00 |
| 431,00 | 269,00 | 50,00 | Apigenin-8-c-glucoside (VITEXIN) | -55,00 | -30,00 |
| 431,00 | 341,00 | 50,00 | Apigenin-8-c-glucoside (VITEXIN) | -55,00 | -30,00 |
| 464,00 | 301,00 | 50,00 | Quercetin-3-O-galactoside | -60,00 | -38,00 |
| 464,00 | 463,00 | 50,00 | Quercetin-3-O-galactoside | -60,00 | -38,00 |
| 594,00 | 593,00 | 50,00 | Kaempferol-3-O-rutinoside | -65,00 | -45,00 |
| 594,00 | 285,00 | 50,00 | Kaempferol-3-O-rutinoside | -65,00 | -45,00 |
| 316,00 | 300,00 | 50,00 | Isorhamnetin | -60,00 | -30,00 |
| 316,00 | 151,00 | 50,00 | Isorhamnetin | -60,00 | -30,00 |
| 316,00 | 315,00 | 50,00 | Isorhamnetin | -60,00 | -30,00 |
| 538,00 | 537,00 | 50,00 | Amentoflavone | -60,00 | -40,00 |
| 538,00 | 375,00 | 50,00 | Amentoflavone | -60,00 | -40,00 |
| 353,00 | 191,00 | 50,00 | Caffeoylquinic acid derivative | -60,00 | -30,00 |
| 353,00 | 179,00 | 50,00 | Caffeoylquinic acid derivative | -60,00 | -30,00 |
| 609,00 | 463,00 | 50,00 | Quercetin rutinoside | -60,00 | -30,00 |
| 609,00 | 300,00 | 50,00 | Quercetin rutinoside | -60,00 | -30,00 |
| 505,00 | 463,00 | 50,00 | Quercetin acetylhexoside | -60,00 | -30,00 |
| 505,00 | 445,00 | 50,00 | Quercetin acetylhexoside | -60,00 | -30,00 |
| 183,00 | 169,00 | 50,00 | Methyl gallate | -60,00 | -20,00 |
| 183,00 | 125,00 | 50,00 | Methyl gallate | -60,00 | -20,00 |
| 593,00 | 425,00 | 50,00 | EGC-epicatechin dimer | -60,00 | -30,00 |
| 197,00 | 169,00 | 50,00 | Ethyl gallate | -60,00 | -20,00 |
| 197,00 | 125,00 | 50,00 | Ethyl gallate | -60,00 | -20,00 |
| 729,00 | 577,00 | 50,00 | Procyanidin dimer gallate | -60,00 | -30,00 |
| 493,00 | 317,00 | 50,00 | Myricetin-3-O-glucuronide | -40,00 | -25,00 |
| 479,00 | 317,00 | 50,00 | Myricetin-3-O-glucoside | -40,00 | -25,00 |
| 441,00 | 289,00 | 50,00 | EC-3-O-gallate | -60,00 | -30,00 |
| 441,00 | 169,00 | 50,00 | EC-3-O-gallate | -60,00 | -30,00 |
| 389,00 | 227,00 | 50,00 | cis-Resveratrol-3-O-glucoside | -40,00 | -25,00 |
| 561,00 | 289,00 | 50,00 | E-fisetinidol-EC isomer 2 | -71,00 | -22,00 |
| 561,00 | 273,00 | 50,00 | E-fisetinidol-EC isomer 3 | -71,00 | -22,00 |
| 745,00 | 457,00 | 50,00 | EGC-EGC gallate | -60,00 | -20,00 |
| 745,00 | 169,00 | 50,00 | EGC-EGC gallate | -60,00 | -20,00 |
| 619,00 | 457,00 | 50,00 | EGC gallate glucoside | -60,00 | -20,00 |
| 619,00 | 305,00 | 50,00 | EGC gallate glucoside | -60,00 | -20,00 |
| 609,00 | 457,00 | 50,00 | EGC digallate | -60,00 | -20,00 |
| 609,00 | 305,00 | 50,00 | EGC digallate | -60,00 | -30,00 |
| 455,00 | 289,00 | 50,00 | EC methyl gallate | -60,00 | -30,00 |
| 455,00 | 183,00 | 50,00 | EC methyl gallate | -60,00 | -30,00 |
| 425,00 | 169,00 | 50,00 | E-Afzelechin gallate | -60,00 | -30,00 |
| 715,00 | 563,00 | 50,00 | Theaflavin gallate | -60,00 | -30,00 |
| 715,00 | 545,00 | 50,00 | Theaflavin gallate | -60,00 | -30,00 |
| 867,00 | 715,00 | 50,00 | Theaflavin diglallate | -60,00 | -30,00 |
| 867,00 | 563,00 | 50,00 | Theaflavin diglallate | -60,00 | -30,00 |
Supplementary Table S2. Precursor ions, product ions, collision energy, and declustering potential parameters of MRM analysis.

## Slide 4
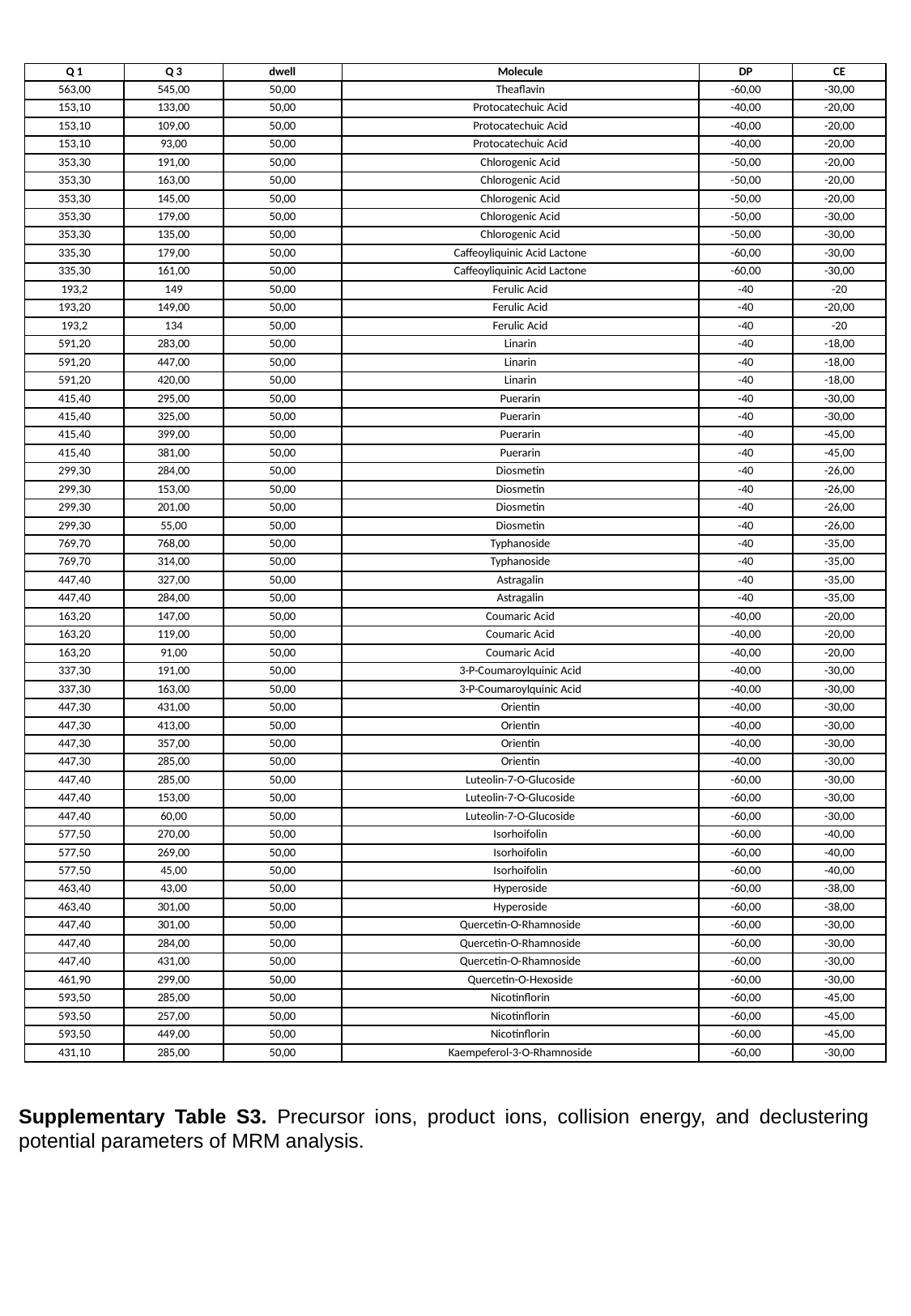

| Q 1 | Q 3 | dwell | Molecule | DP | CE |
| --- | --- | --- | --- | --- | --- |
| 563,00 | 545,00 | 50,00 | Theaflavin | -60,00 | -30,00 |
| --- | --- | --- | --- | --- | --- |
| 153,10 | 133,00 | 50,00 | Protocatechuic Acid | -40,00 | -20,00 |
| 153,10 | 109,00 | 50,00 | Protocatechuic Acid | -40,00 | -20,00 |
| 153,10 | 93,00 | 50,00 | Protocatechuic Acid | -40,00 | -20,00 |
| 353,30 | 191,00 | 50,00 | Chlorogenic Acid | -50,00 | -20,00 |
| 353,30 | 163,00 | 50,00 | Chlorogenic Acid | -50,00 | -20,00 |
| 353,30 | 145,00 | 50,00 | Chlorogenic Acid | -50,00 | -20,00 |
| 353,30 | 179,00 | 50,00 | Chlorogenic Acid | -50,00 | -30,00 |
| 353,30 | 135,00 | 50,00 | Chlorogenic Acid | -50,00 | -30,00 |
| 335,30 | 179,00 | 50,00 | Caffeoyliquinic Acid Lactone | -60,00 | -30,00 |
| 335,30 | 161,00 | 50,00 | Caffeoyliquinic Acid Lactone | -60,00 | -30,00 |
| 193,2 | 149 | 50,00 | Ferulic Acid | -40 | -20 |
| 193,20 | 149,00 | 50,00 | Ferulic Acid | -40 | -20,00 |
| 193,2 | 134 | 50,00 | Ferulic Acid | -40 | -20 |
| 591,20 | 283,00 | 50,00 | Linarin | -40 | -18,00 |
| 591,20 | 447,00 | 50,00 | Linarin | -40 | -18,00 |
| 591,20 | 420,00 | 50,00 | Linarin | -40 | -18,00 |
| 415,40 | 295,00 | 50,00 | Puerarin | -40 | -30,00 |
| 415,40 | 325,00 | 50,00 | Puerarin | -40 | -30,00 |
| 415,40 | 399,00 | 50,00 | Puerarin | -40 | -45,00 |
| 415,40 | 381,00 | 50,00 | Puerarin | -40 | -45,00 |
| 299,30 | 284,00 | 50,00 | Diosmetin | -40 | -26,00 |
| 299,30 | 153,00 | 50,00 | Diosmetin | -40 | -26,00 |
| 299,30 | 201,00 | 50,00 | Diosmetin | -40 | -26,00 |
| 299,30 | 55,00 | 50,00 | Diosmetin | -40 | -26,00 |
| 769,70 | 768,00 | 50,00 | Typhanoside | -40 | -35,00 |
| 769,70 | 314,00 | 50,00 | Typhanoside | -40 | -35,00 |
| 447,40 | 327,00 | 50,00 | Astragalin | -40 | -35,00 |
| 447,40 | 284,00 | 50,00 | Astragalin | -40 | -35,00 |
| 163,20 | 147,00 | 50,00 | Coumaric Acid | -40,00 | -20,00 |
| 163,20 | 119,00 | 50,00 | Coumaric Acid | -40,00 | -20,00 |
| 163,20 | 91,00 | 50,00 | Coumaric Acid | -40,00 | -20,00 |
| 337,30 | 191,00 | 50,00 | 3-P-Coumaroylquinic Acid | -40,00 | -30,00 |
| 337,30 | 163,00 | 50,00 | 3-P-Coumaroylquinic Acid | -40,00 | -30,00 |
| 447,30 | 431,00 | 50,00 | Orientin | -40,00 | -30,00 |
| 447,30 | 413,00 | 50,00 | Orientin | -40,00 | -30,00 |
| 447,30 | 357,00 | 50,00 | Orientin | -40,00 | -30,00 |
| 447,30 | 285,00 | 50,00 | Orientin | -40,00 | -30,00 |
| 447,40 | 285,00 | 50,00 | Luteolin-7-O-Glucoside | -60,00 | -30,00 |
| 447,40 | 153,00 | 50,00 | Luteolin-7-O-Glucoside | -60,00 | -30,00 |
| 447,40 | 60,00 | 50,00 | Luteolin-7-O-Glucoside | -60,00 | -30,00 |
| 577,50 | 270,00 | 50,00 | Isorhoifolin | -60,00 | -40,00 |
| 577,50 | 269,00 | 50,00 | Isorhoifolin | -60,00 | -40,00 |
| 577,50 | 45,00 | 50,00 | Isorhoifolin | -60,00 | -40,00 |
| 463,40 | 43,00 | 50,00 | Hyperoside | -60,00 | -38,00 |
| 463,40 | 301,00 | 50,00 | Hyperoside | -60,00 | -38,00 |
| 447,40 | 301,00 | 50,00 | Quercetin-O-Rhamnoside | -60,00 | -30,00 |
| 447,40 | 284,00 | 50,00 | Quercetin-O-Rhamnoside | -60,00 | -30,00 |
| 447,40 | 431,00 | 50,00 | Quercetin-O-Rhamnoside | -60,00 | -30,00 |
| 461,90 | 299,00 | 50,00 | Quercetin-O-Hexoside | -60,00 | -30,00 |
| 593,50 | 285,00 | 50,00 | Nicotinflorin | -60,00 | -45,00 |
| 593,50 | 257,00 | 50,00 | Nicotinflorin | -60,00 | -45,00 |
| 593,50 | 449,00 | 50,00 | Nicotinflorin | -60,00 | -45,00 |
| 431,10 | 285,00 | 50,00 | Kaempeferol-3-O-Rhamnoside | -60,00 | -30,00 |
Supplementary Table S3. Precursor ions, product ions, collision energy, and declustering potential parameters of MRM analysis.

## Slide 5
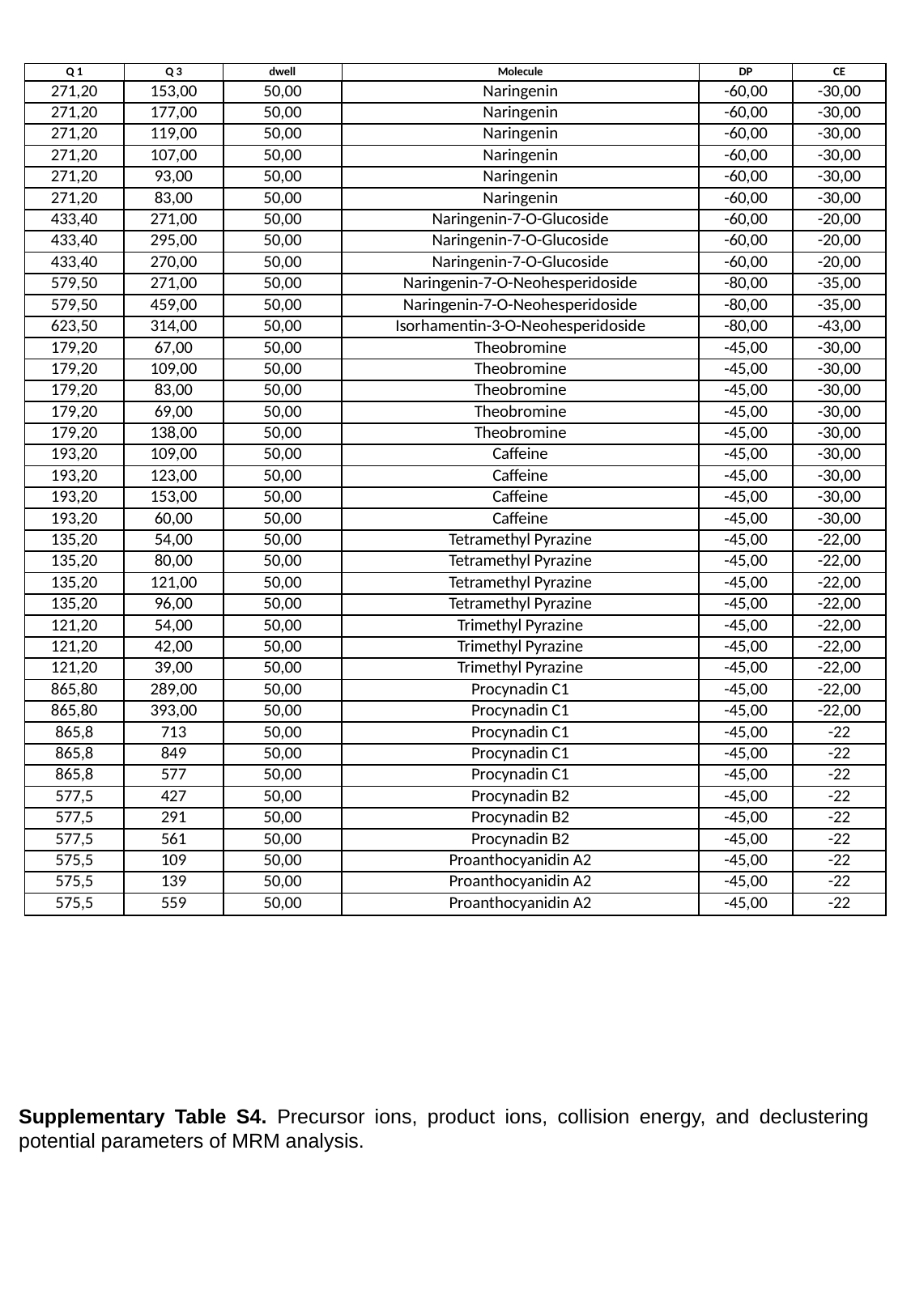

| Q 1 | Q 3 | dwell | Molecule | DP | CE |
| --- | --- | --- | --- | --- | --- |
| 271,20 | 153,00 | 50,00 | Naringenin | -60,00 | -30,00 |
| --- | --- | --- | --- | --- | --- |
| 271,20 | 177,00 | 50,00 | Naringenin | -60,00 | -30,00 |
| 271,20 | 119,00 | 50,00 | Naringenin | -60,00 | -30,00 |
| 271,20 | 107,00 | 50,00 | Naringenin | -60,00 | -30,00 |
| 271,20 | 93,00 | 50,00 | Naringenin | -60,00 | -30,00 |
| 271,20 | 83,00 | 50,00 | Naringenin | -60,00 | -30,00 |
| 433,40 | 271,00 | 50,00 | Naringenin-7-O-Glucoside | -60,00 | -20,00 |
| 433,40 | 295,00 | 50,00 | Naringenin-7-O-Glucoside | -60,00 | -20,00 |
| 433,40 | 270,00 | 50,00 | Naringenin-7-O-Glucoside | -60,00 | -20,00 |
| 579,50 | 271,00 | 50,00 | Naringenin-7-O-Neohesperidoside | -80,00 | -35,00 |
| 579,50 | 459,00 | 50,00 | Naringenin-7-O-Neohesperidoside | -80,00 | -35,00 |
| 623,50 | 314,00 | 50,00 | Isorhamentin-3-O-Neohesperidoside | -80,00 | -43,00 |
| 179,20 | 67,00 | 50,00 | Theobromine | -45,00 | -30,00 |
| 179,20 | 109,00 | 50,00 | Theobromine | -45,00 | -30,00 |
| 179,20 | 83,00 | 50,00 | Theobromine | -45,00 | -30,00 |
| 179,20 | 69,00 | 50,00 | Theobromine | -45,00 | -30,00 |
| 179,20 | 138,00 | 50,00 | Theobromine | -45,00 | -30,00 |
| 193,20 | 109,00 | 50,00 | Caffeine | -45,00 | -30,00 |
| 193,20 | 123,00 | 50,00 | Caffeine | -45,00 | -30,00 |
| 193,20 | 153,00 | 50,00 | Caffeine | -45,00 | -30,00 |
| 193,20 | 60,00 | 50,00 | Caffeine | -45,00 | -30,00 |
| 135,20 | 54,00 | 50,00 | Tetramethyl Pyrazine | -45,00 | -22,00 |
| 135,20 | 80,00 | 50,00 | Tetramethyl Pyrazine | -45,00 | -22,00 |
| 135,20 | 121,00 | 50,00 | Tetramethyl Pyrazine | -45,00 | -22,00 |
| 135,20 | 96,00 | 50,00 | Tetramethyl Pyrazine | -45,00 | -22,00 |
| 121,20 | 54,00 | 50,00 | Trimethyl Pyrazine | -45,00 | -22,00 |
| 121,20 | 42,00 | 50,00 | Trimethyl Pyrazine | -45,00 | -22,00 |
| 121,20 | 39,00 | 50,00 | Trimethyl Pyrazine | -45,00 | -22,00 |
| 865,80 | 289,00 | 50,00 | Procynadin C1 | -45,00 | -22,00 |
| 865,80 | 393,00 | 50,00 | Procynadin C1 | -45,00 | -22,00 |
| 865,8 | 713 | 50,00 | Procynadin C1 | -45,00 | -22 |
| 865,8 | 849 | 50,00 | Procynadin C1 | -45,00 | -22 |
| 865,8 | 577 | 50,00 | Procynadin C1 | -45,00 | -22 |
| 577,5 | 427 | 50,00 | Procynadin B2 | -45,00 | -22 |
| 577,5 | 291 | 50,00 | Procynadin B2 | -45,00 | -22 |
| 577,5 | 561 | 50,00 | Procynadin B2 | -45,00 | -22 |
| 575,5 | 109 | 50,00 | Proanthocyanidin A2 | -45,00 | -22 |
| 575,5 | 139 | 50,00 | Proanthocyanidin A2 | -45,00 | -22 |
| 575,5 | 559 | 50,00 | Proanthocyanidin A2 | -45,00 | -22 |
Supplementary Table S4. Precursor ions, product ions, collision energy, and declustering potential parameters of MRM analysis.
